# Supplementary material for: Differential Role of Phosphorylation in Glucagon Family Receptor Signaling Revealed by Mass Spectrometry
Source: J Proteome Res. 2025 Jun 12;24(7):3367–78. doi: 10.1021/acs.jproteome.5c00079 (PMC12235690; doi:10.1021/acs.jproteome.5c00079)
Supplement: Supplementary file 1 [file pr5c00079_si_001.pdf]

Supporting information for:

Differential role of phosphorylation in glucagon family receptor signaling revealed by mass spectrometry

[1#]Ian M. Lamb, [1#]Alex D. White, [1*]Francis S. Willard, [1]Michael J. Chalmers, [1*]Junpeng Xiao.

[1]Molecular Pharmacology, Discovery Chemistry Research and Technologies, Eli Lilly and Company, Indianapolis, IN, 46285, United States.

[#]These authors contributed equally.

[*]Corresponding authors: Francis S. Willard, Junpeng Xiao.

Email: willardfs@lilly.com, xiao_junpeng@lilly.com

Table of Contents

Figure S1. Enrichment of GCGR using biotinylated peptide ligands and bottom-up proteomic analysis of C-tail phosphorylation (**Page S3**).

Figure S2. Bottom-up proteomic analysis of GLP-1R C-tail phosphorylation (**Page S5**).

Figure S3. Extracted ion chromatograms of C-tail proteoforms of GLP-1R_TEV following GLP-1 treatment (**Page S6**).

Figure S4a-e. Localization of sites of phosphorylation within the GLP-1R_TEV, GIPR_TEV, and GCGR_TEV C-tail. (**Page S7-S12**)

Figure S5. Middle-down proteomic analysis of C-tail phosphorylation in GLP-1R_TEV S442A/S444A/S445A mutant. (**Page S13**)

Figure S6. Middle-down proteomic analysis of C-tail phosphorylation in GCGR_TEV and GIPR_TEV receptors. (**Page S14**)

Figure S7. Extracted ion chromatograms of the GCGR_TEV C-tail proteoforms following GCG treatment. (**Page S15**)

Figure S8. Extracted ion chromatograms of GIPR_TEV C-tail proteoforms following GIP treatment. (**Page S16**)

Figure S9. Receptors with TEV-insertions retain functionality. (**Page S17**)

Figure S10. Ligand induced internalization of GLP-1R phosphorylation site mutants. (**Page S18**)

Table S1. Pharmacological parameters for cAMP accumulation and β-arrestin recruitment assays. (**Page S20**)

Table S2. Identities of raw mass spectrometry files uploaded to https://repository.jpostdb.org/ (jPOSTrepo). (**Page S22**)

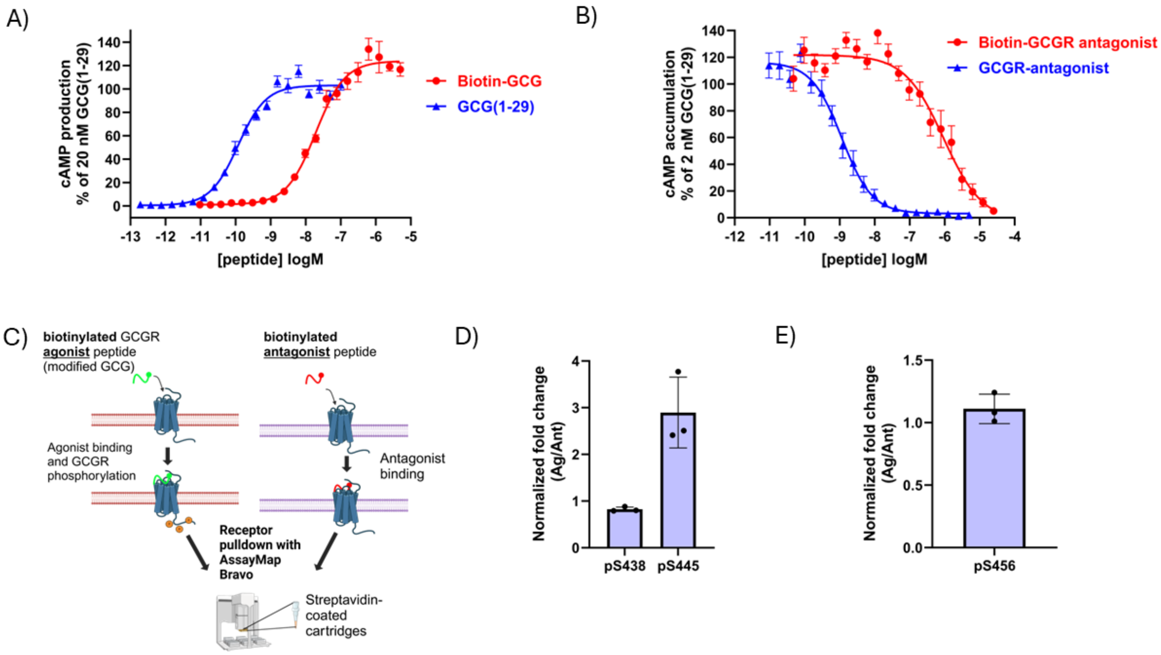

Figure S1. Enrichment of GCGR using biotinylated peptide ligands and bottom-up proteomic analysis of C-tail phosphorylation. A-B) GCGR cAMP accumulation was measured in (A) agonist mode and (B) antagonist mode using native and biotinylated peptides. Summary statistics for pharmacological data are presented in Table S1. C) Schematic diagram of method for enrichment of agonist and antagonist-bound GCGR for proteomic analysis. 1 μM of each ligand was used for enrichment. D-E) Quantification of C-tail phosphorylation of agonist-bound receptor relative to antagonist-bound receptor at the three residues detected to be phosphorylated. Normalization was done to account for the efficiency difference of enrichment between biotinylated agonist vs. antagonist. For each peptide containing a phosphorylated residue, normalization was done by summing the peak area for all proteoforms (modified and unmodified) from all samples in each treatment group (agonist vs. antagonist treated) to get a grand total peak area for agonist treated samples and for antagonist treated samples. For each individual sample, the peak area of each proteoform was divided by the agonist or antagonist grand total peak area to obtain the percentage abundance, which was used to calculate the fold change (agonist/antagonist) as a function of percent

abundance of that proteoform. Data points and error bars are the mean and SD of averaged technical quadruplicates from three independent experiments.

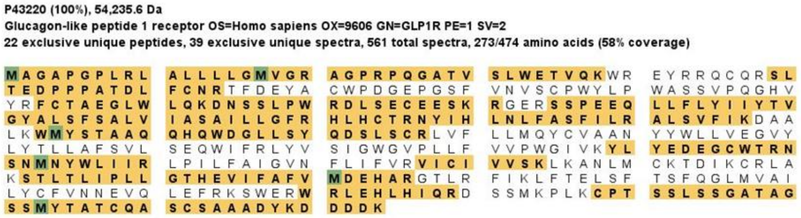

Figure S2. Bottom-up proteomic analysis of GLP-1R C-tail phosphorylation. Graphical display of sequence coverage of GLP-1R-FLAG proteomic analysis. Peptides detected by LC-MS/MS are highlighted in yellow; modified amino acids are shown in green. Note the absence of serine or threonine phosphorylation in the C-tail or anywhere on the receptor (no green “S” or “T”).

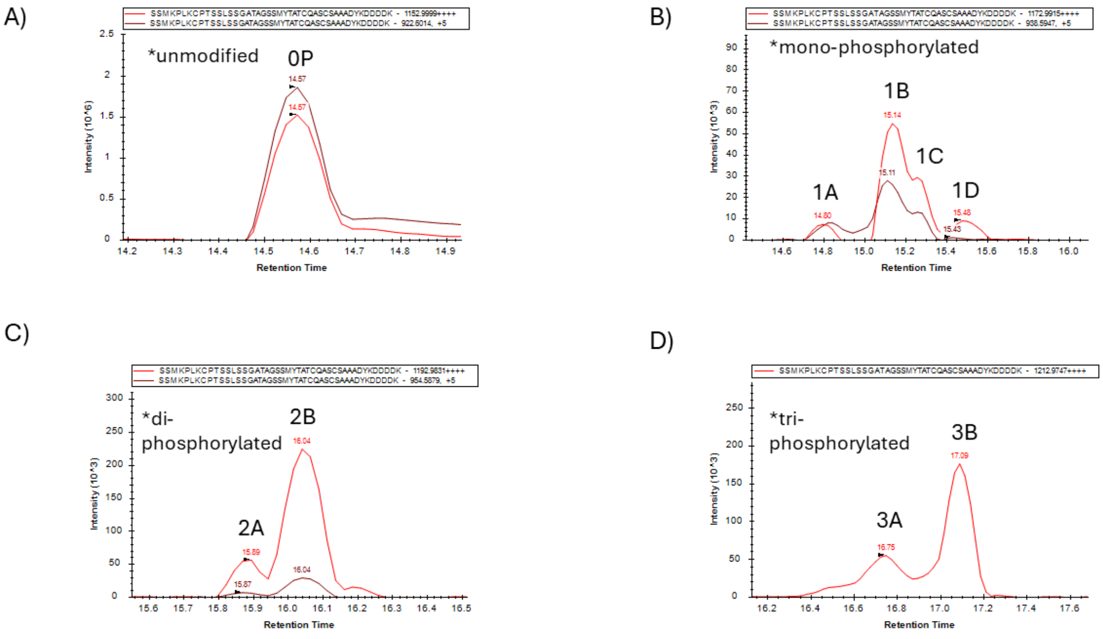

Figure S3. Extracted ion chromatograms of C-tail proteoforms of GLP-1R_TEV following GLP-1 treatment. The peptide analyte and m/z ratio (Th) of the precursor ions are shown in a legend above each panel. Ion intensity corresponds to the sum of the y_3, y_4, y_5, y_6, y_7, y_8, y_9, y_{10} product ions. 0P= unmodified peptide; 1A-1D= four different proteoforms of mono-phosphorylated analyte; 2A-2B= two different proteoforms of di-phosphorylated analyte; 3A-3B= two different proteoforms of tri-phosphorylated analyte.

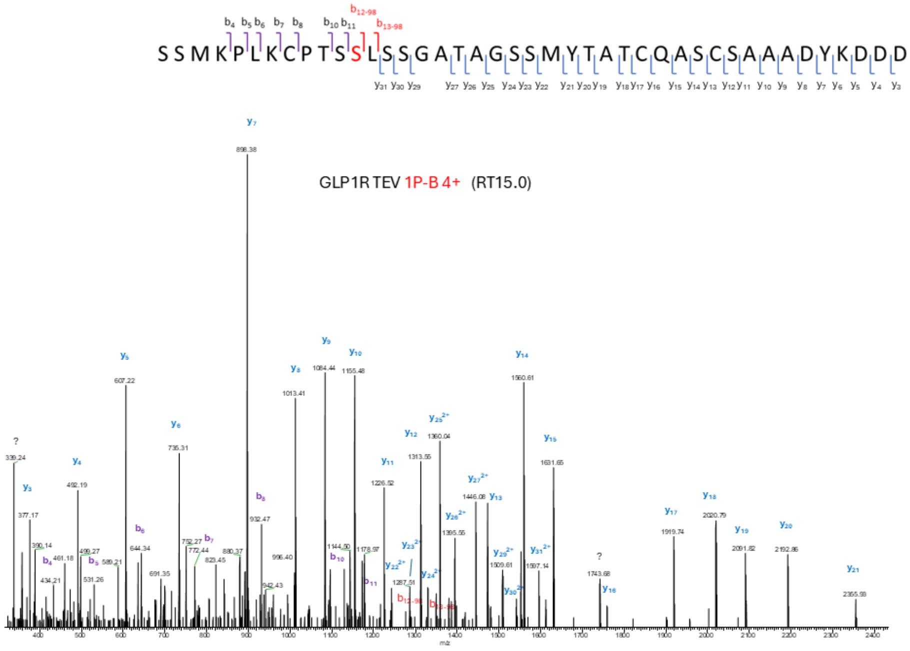

Figure S4a. Localization of sites of phosphorylation within the GLP-1R_TEV C-tail. Sites of phosphorylation within the C-tail peptides were localized from inspection of the product ion spectra for each proteoform. For example, upon dissociation of the $[M+4H]^{4+}$ precursor ion corresponding to GLP-1R_TEV proteoform 1P-B (shown) the MS/MS data yielded extensive b and y product ions. Location of the phosphorylation to the serine in position 12 (S442) was evidenced from the detection of (1) the non-phosphorylated b_{11} and y_{31}^{2+} ions and (2) the detection of the $b_{12\text{-}98}$ and $b_{13\text{-}98}$ ions. For certain proteoforms

we lacked adequate sequence specific product ions to localize the phosphorylation to a single amino acid. In these cases, we report the minimal possible sites of modification based on the information available.

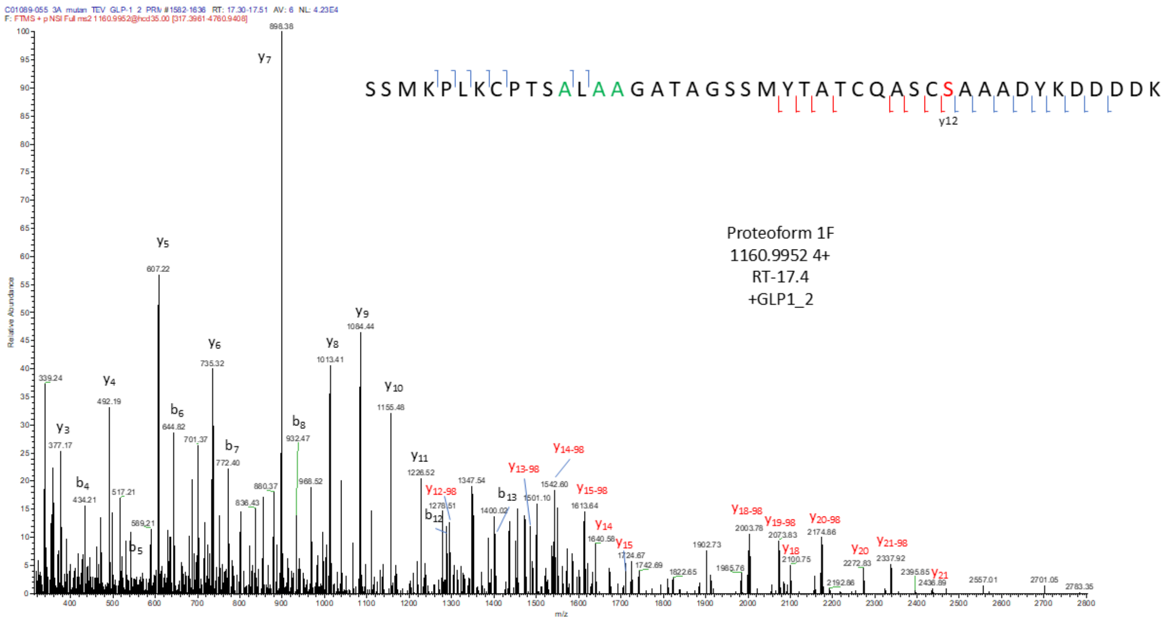

Figure S4b. Localization of sites of phosphorylation within the GLP-1R_TEV 3S to A C-tail. Sites of phosphorylation within the C-tail of the "3S to A" mutant GLP-1R. Upon dissociation of the $[M+4H]^{4+}$ precursor ion corresponding to GLP-1R_TEV proteoform 1P-F (shown in Fig S5D) the MS/MS data yielded extensive b and y product ions localizing the phosphorylated serine to position 33 (S463).

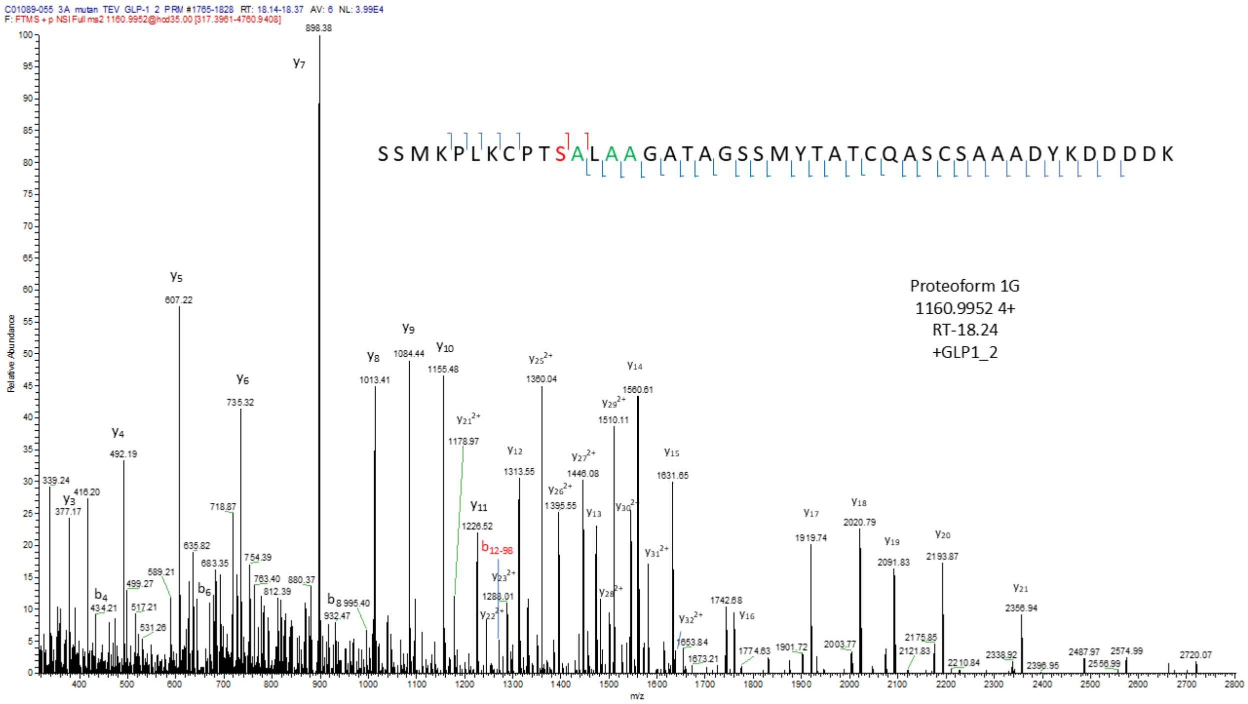

Figure S4c. Localization of sites of phosphorylation within the GLP-1R_TEV 3S to A C-tail. Sites of phosphorylation within the C-tail of the "3S to A" GLP1R mutant. Upon dissociation of the $[M+4H]^{4+}$ precursor ion corresponding to GLP-1R_TEV proteoform 1P-G (shown in Fig S5D) the MS/MS data yielded extensive b and y product ions localizing the phosphorylated serine to position 11 (S441).

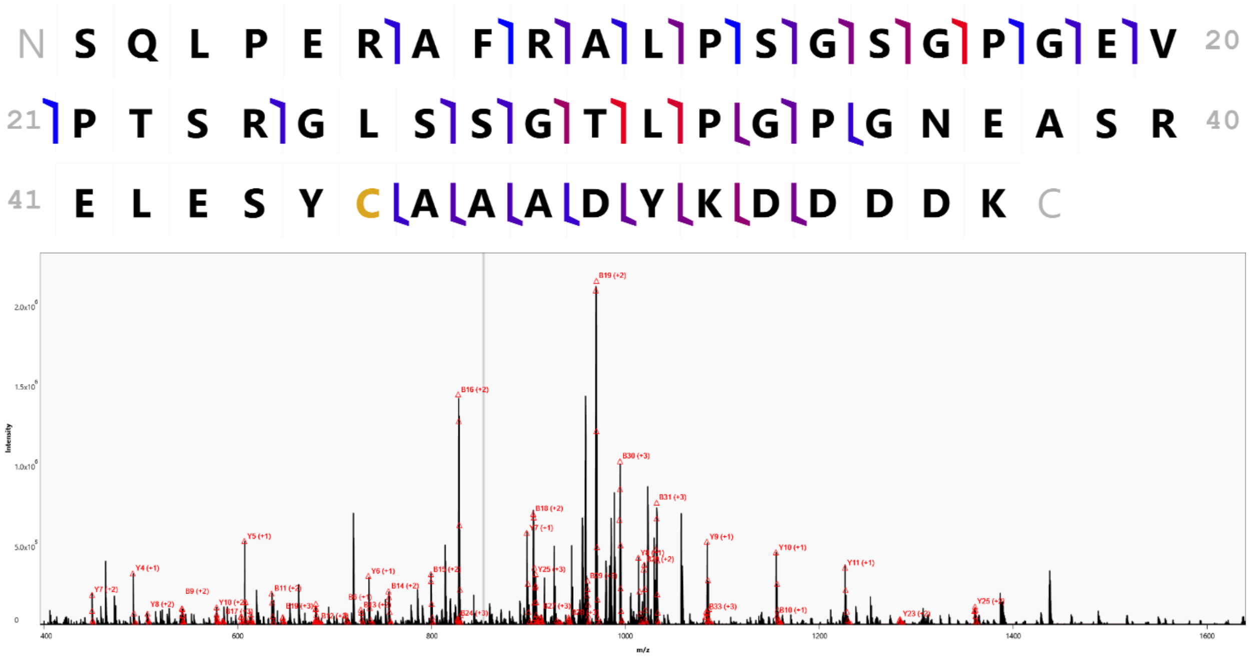

Figure S4d. GIPR_TEV C-tail. Upon dissociation of the $[M+7H]^{7+}$ precursor ion corresponding to GIPR_TEV 0P proteoform the MS/MS data yielded extensive b and y product ions. Images below generated from ProSite TopDown Validator.

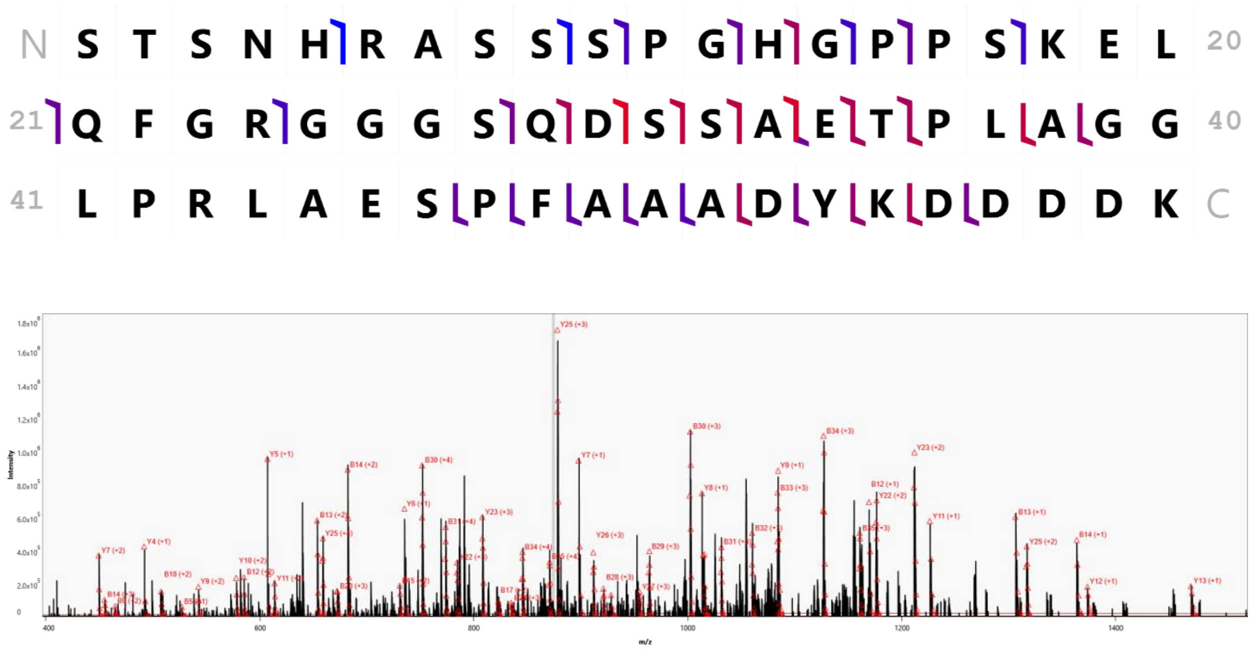

Figure S4e. GCGR_TEV C-tail. Upon dissociation of the $[M+7H]^{7+}$ precursor ion corresponding to GCGR_TEV 0P proteoform the MS/MS data yielded extensive b and y product ions. Images below generated from ProSite TopDown Validator.

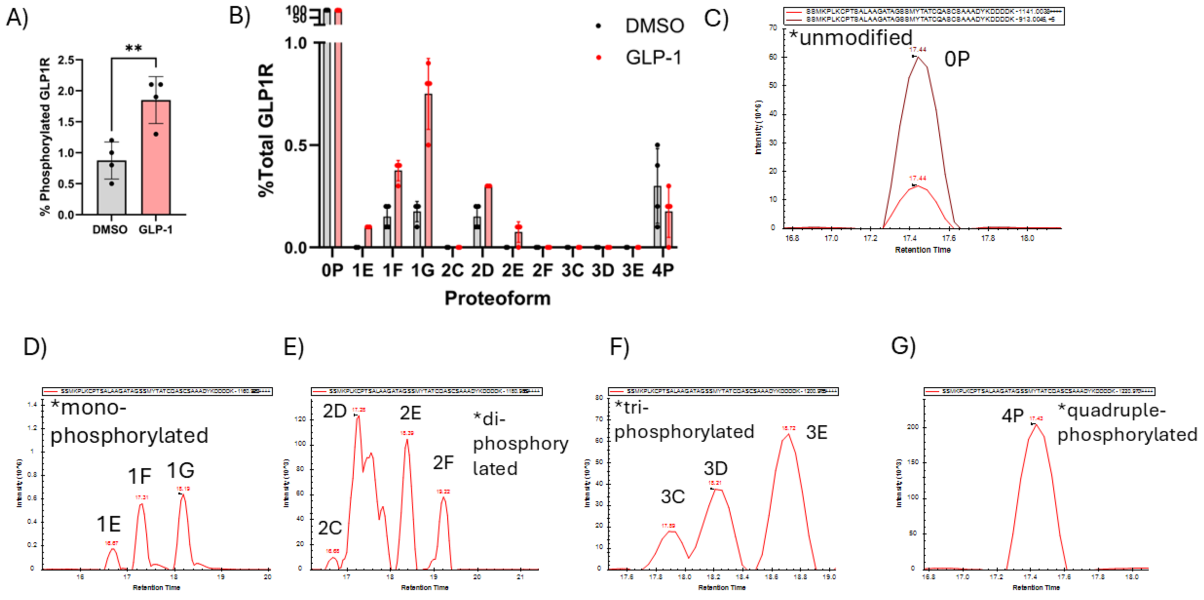

Figure S5. Middle-down proteomic analysis of C-tail phosphorylation in GLP-1R_TEV S442A/S444A/S445A mutant. A) Bar graph indicating the percentage of the GLP-1R_TEV S442A/S444A/S445A mutant C-tail that is phosphorylated following treatment with 1µM GLP-1(7-36). Data points and error bars are the mean and SD of technical quadruplicates from a representative example of three independent experiments. An unpaired, two-tail t-test was conducted for statistical analysis; $^{**}P < 0.01$. B) Bar graph indicating the percentage of each discrete proteoform among total GLP-1R_TEV S442A/S444A/S445A triple mutant receptor after treatment with DMSO (grey bars) or GLP-1(7-36) (red bars). 0P is an unmodified GLP-1R S442A/S444A/S445A proteoform; 1E-1G are mono-phosphorylated proteoforms; 2C-2F are di-phosphorylated proteoforms; 3C-3E are tri-phosphorylated proteoforms; 4P is a quadruple-phosphorylated proteoform. C-G) Extracted ion chromatograms of the proteoforms of the GLP-1R_TEV triple mutant that are shown in supplemental Figure 5B. The peptide analyte and m/z ratio (Th) of the precursor ions are shown in a legend above each panel. Ion intensity corresponds to the sum of the y_3, y_4, y_5, y_6, y_7, y_8, y_9, y_{10}, y_{11} product ions.

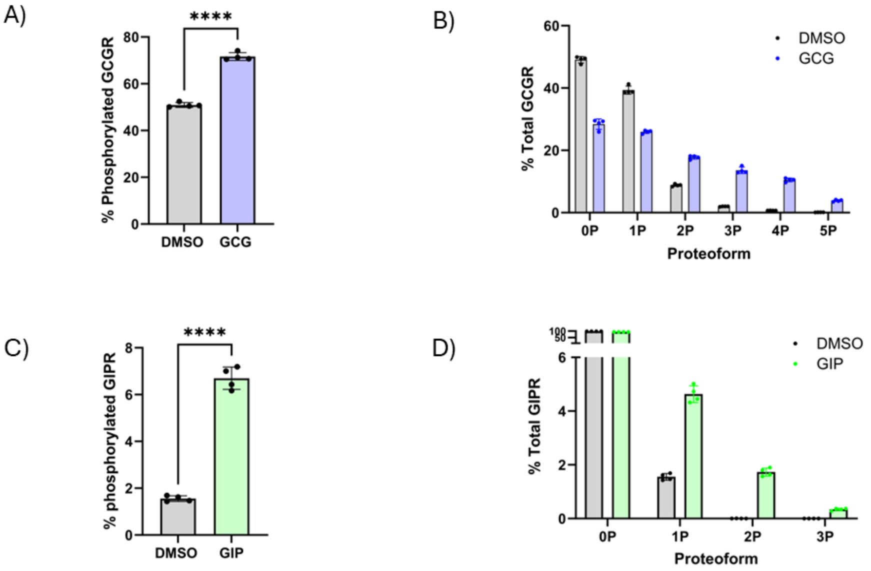

Figure S6. Middle-down proteomic analysis of C-tail phosphorylation in GCGR_TEV and GIPR_TEV receptors. A) Bar graph indicating the percentage of the GCGR_TEV C-tail that is phosphorylated following treatment with 1 µM GCG (1-29). B) Bar graph indicating the percentage of each discrete proteoform among total GCGR_TEV receptor after treatment either 1 µM GCG (1-29) (blue bars) or DMSO (grey bars). 0P is an unmodified proteoform; 1P is a mono-phosphorylated proteoform; 2P is a di-phosphorylated proteoform; 3P is a tri-phosphorylated proteoform; 4P is a quadruple-phosphorylated proteoform; 5P is a penta-phosphorylated proteoform. C) Bar graph indicating the percentage of GIPR_TEV C-tail that is phosphorylated following treatment with 1 µM GIP (1-42). D) Bar graph indicating the percent abundance of each discrete receptor C-tail proteoform among total GIPR_TEV receptor in cells treated with GIP (1-42) (green bars) or DMSO (grey bars). For each graph for receptor (GCGR_TEV and GIPR_TEV), data points and error bars are the mean and SD of technical quadruplicates from a representative example of three independent experiments. An unpaired, two-tail t test was conducted for statistical analysis; ****$P < 0.0001$.

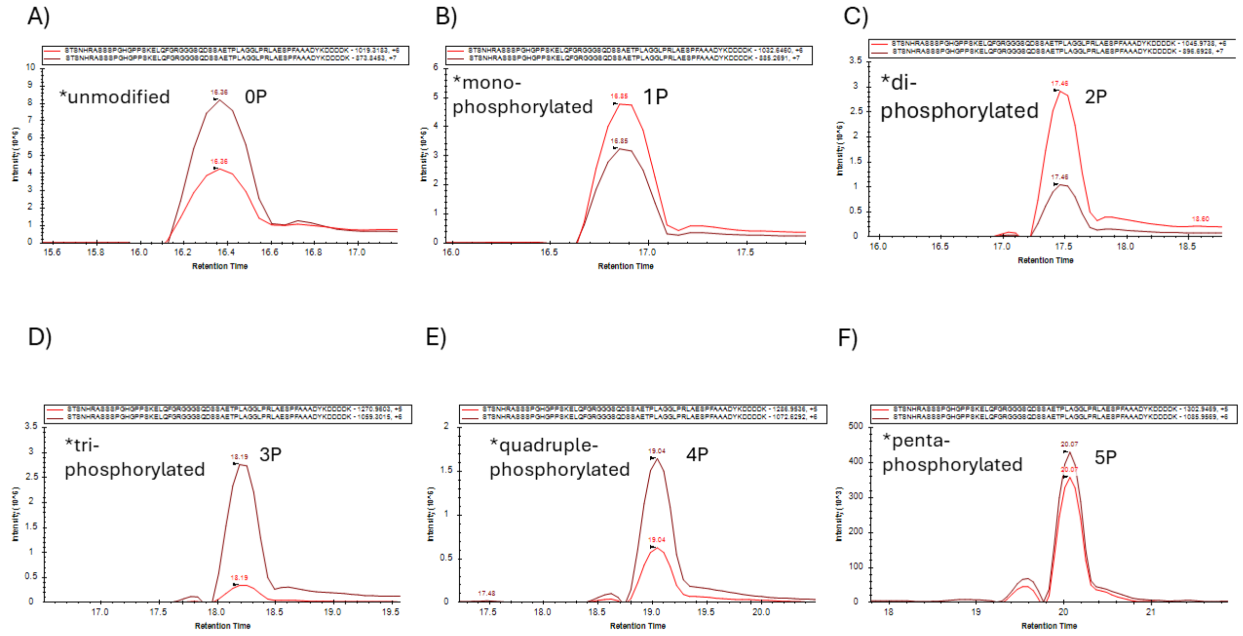

Figure S7. Extracted ion chromatograms of the GCGR_TEV C-tail proteoforms following GCG treatment. The peptide analyte and m/z ratio (Th) of the precursor ions are shown in a legend above each panel. Ion intensity corresponds to the sum of the y_4, y_5, y_6, y_7, y_8, y_9, y_{10}, y_{11} product ions.

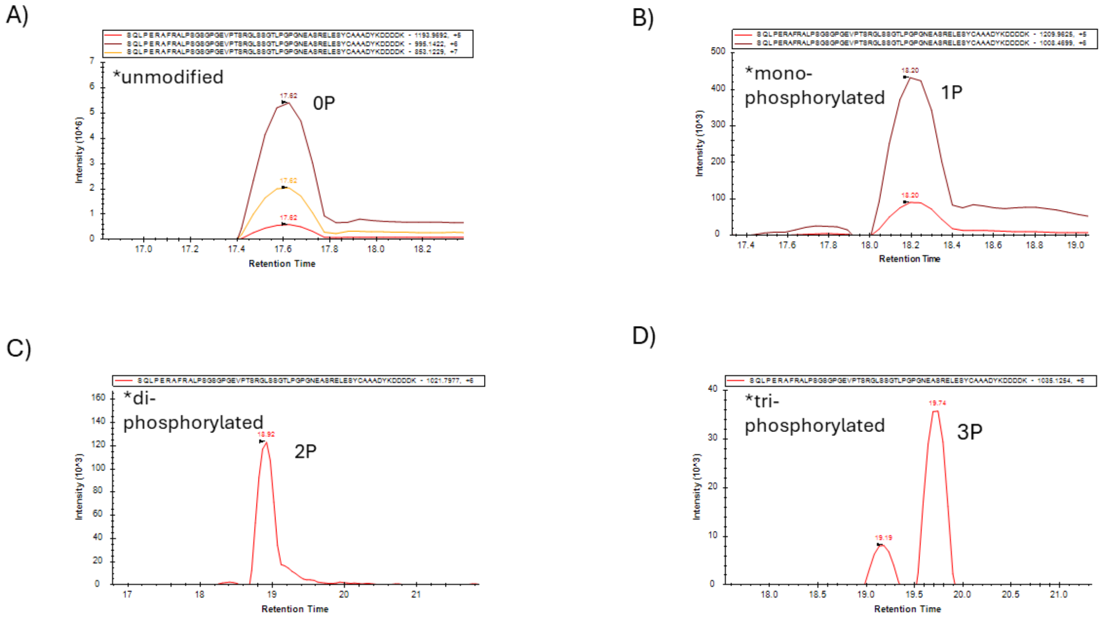

Figure S8. Extracted ion chromatograms of GIPR_TEV C-tail proteoforms following GIP treatment. The peptide analyte and m/z ratio (Th) of the precursor ions are shown in a legend above each panel. Ion intensity corresponds to the sum of the y_4, y_5, y_6, y_7, y_8, y_9, y_{10}, y_{11} product ions.

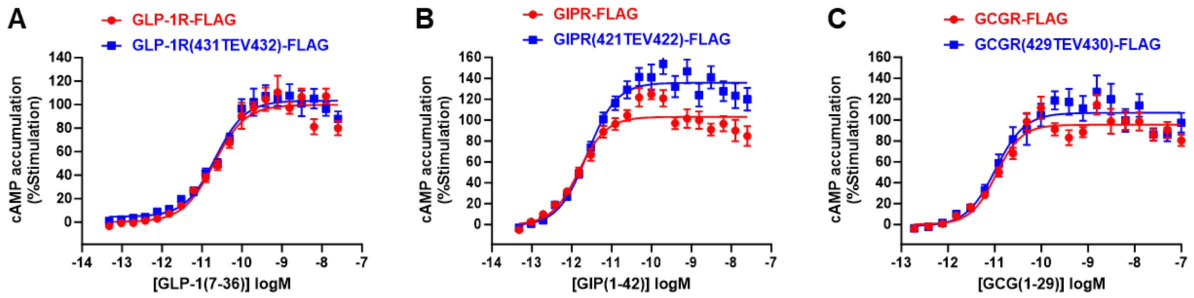

Figure S9. Receptors with TEV-insertions retain functionality. Agonist induced cAMP accumulation for Wild Type and TEV-insertion GLP-1 (A), GIP (B), and GCG (C) receptors was measured. Data shown are the mean of 3 independent experiments. Summary statistics are presented in Table S1.

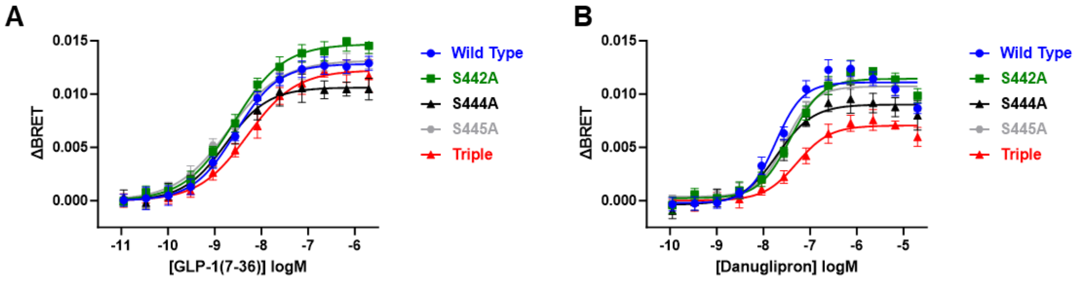

C

		EC50 (nM)	SEM	% Top	SEM	n
Wild Type	GLP-1(7-36)	1.83	0.42	100.0	0.0	3
	Danuglipron	16.19	2.47	88.8	14.7	3
S442A	GLP-1(7-36)	3.90	1.10	101.0	7.7	3
	Danuglipron	18.82	7.78	87.9	13.3	3
S444A	GLP-1(7-36)	3.27	1.45	73.9	5.5	3
	Danuglipron	13.44	3.55	70.3	2.6	3
S445A	GLP-1(7-36)	3.07	1.00	90.2	6.2	3
	Danuglipron	22.37	5.42	78.1	6.0	3
Triple	GLP-1(7-36)	4.82	0.53	103.5	4.9	3
	Danuglipron	21.33	9.50	56.9	4.4	3

Figure S10. Ligand induced internalization of GLP-1R phosphorylation site mutants. GLP-1R internalization in response to GLP-1(7-36) (A) or danuglipron (B) using a bystander BRET-based approach in cells transiently expressing nano-Luc FYVE and wildtype and mutant forms of Halo-tagged GLP1R. Triple denotes the S442A + S444A + S445A cluster mutant. Dose response curves were fit using the 4-parameter logistic equation. Data are the mean of 4-8 replicates from a single experiment. (C) Summary statistics from 3 independent experiments are presented. Potency values are expressed as geometric means and standard errors and efficacy values (%Top; normalized to Wild Type receptor with GLP-1(7-36)) are expressed as arithmetic means and standard errors.

GLP1R	cAMP EC_{50} (nM)		cAMP E_{max}		βarr-2 EC_{50} (nM)		βarr-2 E_{max}	
	GeoMean	Geo SEM	Mean	SEM	GeoMean	Geo SEM	Mean	SEM
**GLP-1(7-36)**								
WT	0.025	0.02	100	0	1.9	0.3	100	0
S442A	0.016	0.008	105	7	1.8	0.3	88	7
S444A	0.026	0.02	107	2	1.6	0.2	94	3
S445A	0.017	0.006	102	3	1.9	0.3	71	7
3S→A	0.008	0.004	108	6	2.0	0.3	51	7
GLP-1R-FLAG	0.032	0.005	100	0				
GLP-1R(431TEV432)-FLAG	0.033	0.005	103	8				
**danuglipron**								
WT	2.0	0.04	99	1	19.7	0.5	100	0
S442A	2.9	1.5	116	10	21	2	84	5
S444A	2.6	0.4	127	7	17.8	0.8	94	4
S445A	3.1	1.0	123	8	21	1	64	6
3S→A	2.1	0.4	130	5	26	2	43	5

GIPR	cAMP EC_{50} (nM)		cAMP E_{max}		βarr-2 EC_{50} (nM)		βarr-2 E_{max}	
	GeoMean	Geo SEM	Mean	SEM	GeoMean	Geo SEM	Mean	SEM
**GIP(1-42)**								
WT	0.014	0.004	97	3	1.3	0.2	100	0
S342A	0.022	0.008	118	8	1.4	0.4	149	1
T343A	0.0022	0.0005	118	7	1.0	0.6	50	7
S415A	0.0064	0.002	147	24	2.4	0.6	60	9
S433A	0.010	0.006	148	10	1.6	0.5	70	10
S447A	0.027	0.04	179	25	1.2	0.5	58	7
S448A	0.011	0.01	157	14	2.3	0.9	56	13
GIPR-FLAG	0.0024	0.0004	100	0				
GIPR(421TEV422)-FLAG	0.0045	0.0011	155	21				

GCGR	Agonist Mode							
	cAMP EC_{50} (nM)		cAMP E_{max}		βarr-2 EC_{50} (nM)		βarr-2 E_{max}	
	GeoMean	Geo SEM	Mean	SEM	GeoMean	Geo SEM	Mean	SEM
**GCG(1-29)**								
WT	0.018	0.00	100	0	6.6	3	100	0
S445A	0.020	0.01	113	7	5.6	3	98	12
S456A	0.015	0.01	132	6	4.7	3	120	11
S459A	0.015	0.003	116	5	4.5	1	125	30
3S→A	0.011	0.0008	123	9	4.8	2	129	17
5S→A	0.0078	0.0020	123	15	5.6	0.4	97	1
GCGR-FLAG	0.0084	0.0029	100	0				
GCGR(429TEV430)-FLAG	0.012	0.0041	119	16				
**GCG(1-29)**	0.16	0.01	103	2				
**Biotin-GCG**	20	1	124	8				

	Antagonist Mode			
	cAMP IC_{50} (nM)		cAMP E_{max}	
	GeoMean	Geo SEM	Mean	SEM
**GCGR Antagonist**	1.2	0.7	116	5
**Biotin-GCGR antagonist**	747	630	120	7

Table S1. Pharmacological parameters for cAMP accumulation and β-arrestin recruitment assays. Dose response curves were fit using the 4-parameter logistic equation. Pharmacological parameters were obtained, and potency values are expressed as geometric means and standard errors and efficacy values (E_{MAX}) are expressed as arithmetic means and standard errors. All data sets are generated from n=3 independent experiments.

Experiment Number	Biological Replicate	Associated Figure
C01089-044	1	supplemental figure 1
C01089-053	2	supplemental figure 1
C01089-054	3	supplemental figure 1
C01089-049	1	figure 2
C01089-050	2	figure 2
C01089-051	3	figure 2
C01089-042	1	figure 3B and D
C01089-056	2	figure 3B and D
C01089-060	3	figure 3B and D
C01089-065	1	figure 3C and E
C01089-066	2	figure 3C and E
C01089-022	1	figure 1
C01089-031	2	figure 1
C01089-036	3	figure 1
C01089-008	1	supplemental figure 2
C01089-055	1	supplemental figure 5
C01089-065	2	supplemental figure 5
C01089-065	3	supplemental figure 5
C01089-059	1	supplemental figure 6A and B
C01089-059	2	supplemental figure 6A and B
C01089-061	3	supplemental figure 6A and B
C01089-058	1	supplemental figure 6C and D
C01089-058	2	supplemental figure 6C and D
C01089-062	3	supplemental figure 6C and D

Table S2. Identities of raw mass spectrometry files uploaded to https://repository.jpostdb.org/ (jPOSTrepo). For the middle-down data, the accession numbers are PXD060260 for ProteomeXchange and JPST003581 for jPOST. For the bottom-up data, the accession numbers are PXD060263 for ProteomeXchange and JPST003580 for jPOST.
